# Prevalence of SARS-CoV-2 specific neutralising antibodies in blood donors from the Lodi Red Zone in Lombardy, Italy, as at 06 April 2020

**DOI:** 10.2807/1560-7917.ES.2020.25.24.2001031

**Published:** 2020-06-18

**Authors:** Elena Percivalle, Giuseppe Cambiè, Irene Cassaniti, Edoardo Vecchio Nepita, Roberta Maserati, Alessandro Ferrari, Raffaella Di Martino, Paola Isernia, Francesco Mojoli, Raffaele Bruno, Marcello Tirani, Danilo Cereda, Carlo Nicora, Massimo Lombardo, Fausto Baldanti

**Affiliations:** 1 Molecular Virology Unit, Microbiology and Virology Department, IRCCS Policlinico San Matteo, Pavia, Italy; 2 Immunohematology and Transfusion Medicine Unit, Ospedale Maggiore di Lodi, Lodi, Italy; 3 Department of Clinical Surgical Diagnostic and Pediatric Sciences, University of Pavia, Pavia, Italy; 4 SIMT, Centro Lavorazione e Validazione, IRCCS Policlinico San Matteo, Pavia, Italy; 5 ICU1 Department of Intensive Medicine, IRCCS Policlinico San Matteo, Pavia, Italy; 6 Infectious Diseases I, Department of Medical Sciences and Infectious Diseases, IRCCS Policlinico San Matteo, Pavia, Italy; 7 Health Protection Agency of Pavia, Department of Hygiene and Preventive Medicine, Pavia, Italy; 8 Lombardy Region, Directorate General for Health, UO Prevenzione, Milan, Italy; 9 Chief Executive Office, IRCCS Policlinico San Matteo, Pavia, Italy; 10 Chief Executive Office, ASST Lodi, Lodi, Italy

**Keywords:** SARS-CoV-2 seroprevalence, Lodi Red Zone, epidemiology, microneutralization assay

## Abstract

We evaluated SARS-CoV-2 RNA and neutralising antibodies in blood donors (BD) residing in the Lodi Red Zone, Italy. Of 390 BDs recruited after 20 February 2020 − when the first COVID-19 case in Lombardy was identified, 91 (23%) aged 19–70 years were antibody positive. Viral RNA was detected in an additional 17 (4.3%) BDs, yielding ca 28% (108/390) with evidence of virus exposure. Five stored samples collected as early as 12 February were seropositive.

From late December 2019, the new severe acute respiratory syndrome virus 2 (SARS-CoV-2), which is responsible coronavirus disease (COVID-19), spread worldwide from China, causing a pandemic [1,2]. In Lombardy, Italy, the first laboratory-confirmed COVID-19 case was identified on 20 February 2020, in Castiglione d’Adda, a municipality in the Lodi province [3]. Prompt and thorough epidemiological investigation led to the detection of 113 additional cases by 23 February thus confirming an ongoing COVID-19 outbreak. On 23 February, a regional and national emergency plan was set up, including the complete lockdown of social and commercial activities in an area of 169 km^2^, referred to as the Lodi Red Zone. The Lodi Red Zone included 10 municipalities (Bertonico, Casalpusterlengo, Castelgerundo, Castiglione d’Adda, Codogno, Fombio, Maleo, San Fiorano, Somaglia, Terranova dei Passerini) and 51,500 inhabitants. It constituted, together with another municipality in the province of Veneto, the first lockdown area in Italy.

In this report, registered blood donors (BD) from the Lodi Red Zone, at the beginning of the outbreak, were investigated for exposure to SARS-CoV-2. In some who showed evidence of infection, as well as in a few COVID-19 convalescent patients, SARS-CoV-2 neutralising antibody titres were estimated.

## Study design

We evaluated the seroprevalence of SARS-CoV-2 infection in BDs living in the Lodi Red Zone. A new rapid microneutralisation assay was employed for this purpose. Subsequent to its appraisal, the assay was used to estimate the proportion of antibody-positive individuals in a sample of BDs enrolled after 20 February 2020. These BDs were also tested in parallel for SARS-CoV-2 RNA by real-time RT-PCR to further inform on their exposure to the virus. Stored BD samples collected from 27 January 2020 to 20 February 2020 were also screened with the microneutralisation assay to check for potential circulation of SARS-CoV-2 in Lombardy prior to the identification of the index case. Moreover, to obtain insight on numbers of potential donors for hyperimmune plasma treatment strategies [4-10], we also estimated SARS-CoV-2 neutralisation titres in the enrolled BDs and in a few samples from COVID-19 convalescent patients.

## Samples to appraise the microneutralisation assay

The SARS-CoV-2 microneutralisation assay was appraised by testing 30 serum samples (21 females and 9 males; median age: 43 years, range: 24–74) stored during the pre-pandemic period (between 2011 and 2013) – including 10 positive for other common coronaviruses (229E, OC43, HKU1, NL63), as well as 40 serum samples obtained in the period 15–30 March 2020 from prospectively enrolled SARS-CoV-2 real-time RT-PCR positive patients (14 females and 26 males; median age: 61 years, range 45–81) during convalescence (median 25 days after first SARS-CoV-2 positive nasal swab; range: 9–44).

## Blood donor enrolment and blood donor samples

In the Lodi Red Zone, a total of 2,272 individuals are registered as BDs, corresponding to 4.4% of total inhabitants (n = 51,500) and 6.9% of those in the 18–70 years age range (n = 32,927). BDs were prospectively enrolled at the Blood Transfusion Centre of the Lodi Hospital. Paired serum samples and nasal swabs were collected from 390 blood donors from 18 March to 6 April 2020. History of symptoms or high-risk contacts during the previous 30 days was recorded.

In addition, stored serum samples from 300 BDs of the Lodi Red Zone collected between 27 January 2020 and the first 20 days of February 2020 (before the diagnosis of the first case of COVID-19 in Italy) were analysed.

## Laboratory assays

An in-house microneutralisation assay adapted to SARS-CoV-2 from a previously reported method was employed [11]. In addition, specific real-time RT-PCRs targeting RNA-dependent RNA polymerase and envelope (E) genes were used to detect the presence of SARS-CoV-2 according to the World Health Organization guidelines [12] and the Corman et al. protocol [13]. Details of the methods and analyses are described in the Supplementary Material.

## Ethical statement

The study was performed according to guidelines of the Institutional Review Board of the Fondazione IRCCS Policlinico San Matteo (protocols no. P-20200035863 and P-20200027987).

## Performance of the microneutralisation test

All 30 samples (100%) collected in the pre-pandemic period were negative for SARS-CoV-2 neutralising antibodies (NT-Abs). Moreover, none of the patients with previous common coronavirus infections tested positive for SARS-CoV-2 NT-Abs. On the other hand, the rate of convalescent COVID-19 patients who were positive for SARS-CoV-2 NT-Abs was 38/40 (95%), while the remaining 2/40 (5%) showed a negative NT-Ab titre (NT-Abs < 1:10). Based on these data, the sensitivity of our assay was 95% and the specificity was 100%.

## Infection in blood donors enrolled after 18 March 2020

**Figure 1:**
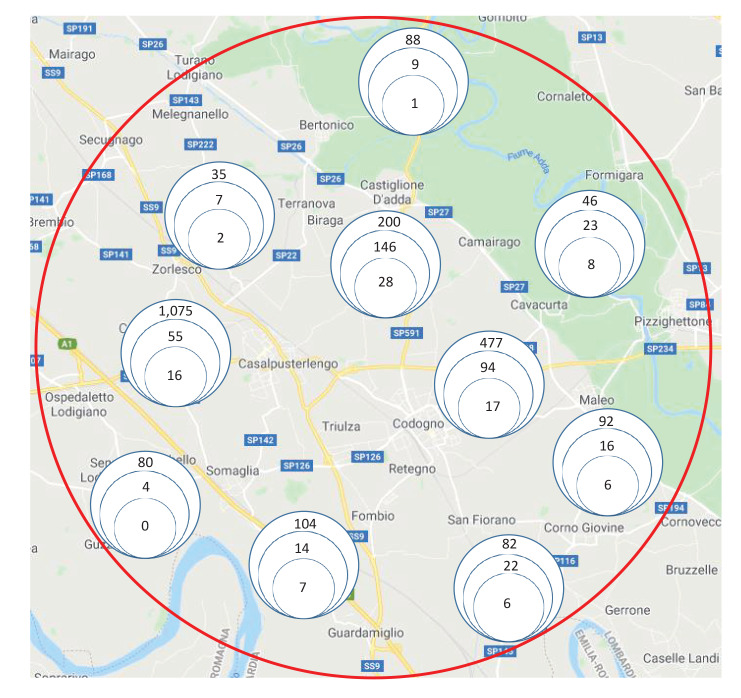
Distribution of blood donors in the 10 municipalities of the Lodi Red Zone, including those recruited for the study and those testing positive for SARS-CoV-2 neutralising antibodies, Lombardy, Italy, 18 March–6 April 2020 (n = 2,272 blood donors)

BD: blood donor; NT-Abs: SARS-CoV-2 neutralising antibodies; SARS-CoV-2: severe acute respiratory syndrome virus 2.

Overall, the 390 BDs recruited between 18 March and 6 April represented 17% of the 2,272 registered BDs residing in the Lodi Red Zone (**Figure 1**). Of these, 118 (30%) were females and 272 (70%) were males. Median age was 46 years (range: 19–70). All the patients were asymptomatic at the time of paired serum and nasal swab sample collection.

The numbers of BDs in each municipality are shown in the outer blue circles. The distribution per municipality of the total 390 BDs recruited for the study and tested for NT-Abs and by nasal swab real-time RT-PCR is reported via the middle circles, while the internal circles indicate the numbers of NT-Ab positive BDs per municipality among the total 91 BDs found positive for NT-Abs. The red circle indicates the limits of the Lodi Red Zone.

Among the 390 BDs, 370 (95%) were negative by SARS-CoV-2 real-time RT-PCR, while 20 tested positive (5%). All 20 SARS-CoV-2 real-time RT-PCR positive BDs were detected between 18 and 20 March. Among them, four reported high-risk contacts with COVID-19 positive patients, five reported mild symptoms during the previous 30 days and three reported both high-risk contacts and mild symptoms. The remaining eight BDs reported that they had neither symptoms nor high-risk contacts (**Table 1** **).**

**Table 1:** COVID-19 related symptoms and risk factors during the 30 days before the sample collection, reported by blood donors testing positive for SARS-CoV-2 RNA in nasal swabs, Lodi Red Zone, Lombardy, Italy, 18–20 March 2020 (n = 20 blood donors)

Symptoms or risk factors	Number
Fever (> 37.5°C)	4
Fatigue	2
Cough	1
Cold	2
Sore throat	1
Anosmia and dysgeusia	3
Muscular pain	1
Diarrhoea	1
High risk contact with COVID-19 positive subjects	7

COVID-19: coronavirus disease; SARS-CoV-2: SARS-CoV-2: severe acute respiratory syndrome virus 2.

The total of the second column is greater than 20 because a given blood donor could have more than one symptom or risk factor.

On the other hand, 91 of 390 (23%) BDs were positive for SARS-CoV-2 specific NT-Abs (≥ 1:10) while 299 (77%) tested negative (< 1:10). The 91 NT-Ab positive samples’ collection dates were distributed over the 18 March to 6 April 2020 period. Only three of 91 (3%) BDs with detectable SARS-CoV-2 NT-Abs were also positive for virus RNA, while most BDs showing positive SARS-CoV-2 NT-Abs (88 of 91; 97%) had no detectable viral RNA at the time of samples collection.

Moreover, 17 of 20 (85%) BDs with SARS-CoV-2 positive real-time RT-PCR had no detectable NT-Abs while the remaining three (15%) had a positive NT-Abs titre against SARS-CoV-2 (**Table 2**). All the 20 real-time RT-PCR positive BDs cleared the virus after 15 days. 

**Table 2:** SARS-CoV-2 real-time RT-PCR and neutralising antibody results in a group of blood donors, Lodi Red Zone, Lombardy, Italy, 18 March–6 April (n = 390 blood donors)

COVID-19	RT-PCR +	RT-PCR −	Total
NT-Abs +	3	88	91
NT-Abs −	17	282	299
Total	20	370	390

COVID-19: coronavirus disease 2019; SARS-CoV-2: severe acute respiratory syndrome virus 2; NT-Abs: neutralising antibodies.

The ‘+’ and ‘−‘ signs indicate if the respective tests were positive or negative.

## Serological results on stored samples obtained before 20 February 2020

Overall, five of 300 (2%) serum samples collected from BDs before 20 February, showed positive NT-Abs against SARS-CoV-2. All the positive samples had been collected between 12 and 17 February. Given the temporal delay between infection and NT-Abs synthesis, it might be hypothesised that the virus circulated well before the detection of the index case. In addition, all the five NT-Ab positive individuals lived in Casalpusterlengo, a town 8.8 km from Castiglione d’Adda.

## Antibody titres in convalescent patients and blood donors

**Figure 2:**
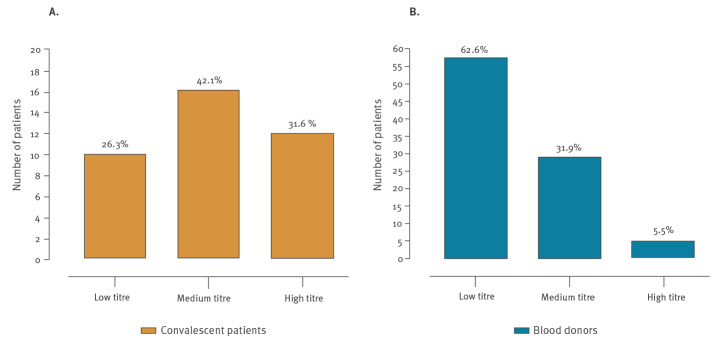
Distribution of NT-Abs titres in convalescent patients and blood donors, Italy, 2011–2020 (n = 129 individuals)

Distribution of the 38 convalescent patients according to NT-Ab titres is shown in Figure 2A. In detail, 10 patients showed a low NT-Ab titre (ranging from 1:10 to 1:40), 16 reported a medium NT-Ab titre (ranging from 1:40 to 1:160) and 12 showed a high NT-Ab titre (higher than 1:160).

Thirty-eight COVID-19 positive patients during convalescence (**A**) and 91 blood donors (**B**) were evaluated.

NT-Abs titres were classified as low (ranging from 1:10 to 1:40), medium (ranging from 1:40 to 1:160) or high (higher than 1:160). Each histogram represents the number of patients for each titre category and all percentages are given in the graph.

Among the 91 BDs with positive NT-Abs, 57 (63%) had low NT-Abs titres (NT-Abs between 1:10 and 1:40), 29 (32%) had medium NT-Abs (NT-Abs between 1:40 and 1:160) while in five (5%) NT-Abs titre was high (NT-Abs higher than 1:160). The distribution of the 91 NT-Ab positive BDs according to NT-Ab titre against SARS-CoV-2 is shown in Figure 2B.

## Discussion

To our knowledge, this is the first investigation on SARS-CoV-2 seroprevalence in asymptomatic individuals in one of the two initial lockdown areas in Northern Italy. In addition, this study reports on NT-Abs titres in convalescent patients with clinical COVID-19 as well as in asymptomatic (or paucisymptomatic) BDs.

Based on our results, only 23% (91/390) of BDs in an area highly affected by the COVID-19 epidemic showed signs of immunological memory to SARS-CoV-2, while presenting with no symptoms or mild symptoms. When taking into account also the real-time RT-PCR-positive BDs, the prevalence of individuals with SARS-CoV-2 infection increased to 28% (108/390). However, this low rate of individuals having already, or (hopefully) soon, SARS-CoV-2 NT-Abs raises the issue of the risk of transmission among the largely susceptible population. Indeed, it could be estimated that by 06 April 2020 as many as 9,087 individuals in the 18–70 years range might have been infected by SARS CoV-2 in the Lodi Red Zone, while almost 24,000 remained susceptible.

Another important observation is that the majority of NT Ab-positive BDs appeared to have lower NT-Ab titres than COVID-19 convalescent patients. Indeed, based on these observations, it could be that the severity of symptoms might be a key determinant for mounting NT-Ab levels. Thus, the treatment with plasma hyperimmune might rely upon the selection of a subset of subjects with high NT-Ab titres.

Finally, in the group of stored plasma samples dating from 27 January–20 February we found ca 2% of samples with evidence of SARS-CoV-2 NT-Abs, suggesting a prior unnoticed circulation of the virus among the population. This might have been favoured by the ongoing influenza season, which could have made mild COVID-19 inconspicuous among all other influenza-like illnesses (ILIs), aside from possibly missing epidemiological links with areas of ongoing transmission. Given that NT-Abs may need time to appear in infected persons, our hypothesis is that the SARS-CoV-2 circulation in Lombardy could have started weeks before the first patient was identified.

When the outbreak was declared, blood and haemocomponent donations were interrupted in the whole Red Zone area from 20 February through 27 April, primarily to limit contact by the donors with the healthcare setting to reduce the infection possibilities.

Additional population studies (including different categories of individuals and other serological assays) are needed to better define the epidemiology of COVID-19. Further investigations are required to determine the role of NT-Abs in the protection against SARS-CoV-2 infection as well as the therapeutic potential of hyperimmune plasma.

## Supplementary Data

474000 Supplementary Material
