# Author's correction for Euro Surveill. 2020;25(24)

**DOI:** 10.2807/1560-7917.ES.2022.27.3.220120c1

**Published:** 2022-01-20

**Authors:** Eurosurveillance editorial team

**Affiliations:** 1 European Centre for Disease Prevention and Control (ECDC), Stockholm, Sweden

In the article ‘Prevalence of SARS-CoV-2 specific neutralising antibodies in blood donors from the Lodi Red Zone in Lombardy, Italy, as at 06 April 2020’ by Percivalle et al., published on 18 June 2020, incorrect protocol numbers were mistakenly quoted in the Ethical statement. The text was corrected on 20 January 2022 at the request of the authors. 

