# Supplementary material for: Prevalence of SARS-CoV-2 specific neutralising antibodies in blood donors from the Lodi Red Zone in Lombardy, Italy, as at 06 April 2020
Source: Euro Surveill. 2020 Jun 18;25(24):2001031. doi: 10.2807/1560-7917.ES.2020.25.24.2001031 (PMC7315724; doi:10.2807/1560-7917.ES.2020.25.24.2001031)
Supplement: Supplementary Material [file 2001031_BALDANTI_Supplementary_Material.pdf]

This supplementary material is hosted by Eurosurveillance as supporting information alongside the article "Prevalence of COVID-19 specific neutralising antibodies in blood donors from the Lodi Red Zone in Lombardy, Italy, as at 06 April 2020", on behalf of the authors, who remain responsible for the accuracy and appropriateness of the content. The same standards for ethics, copyright, attributions and permissions as for the article apply. Supplements are not edited by Eurosurveillance and the journal is not responsible for the maintenance of any links or email addresses provided therein.

Supplementary material

SARS-CoV2 microneutralization assay

Neutralizing antibodies (NT-Abs) titers against SARS-CoV2 was defined following in principle a reported protocol [1]. Briefly, 50 µl of sample from each patient, starting from 1:10 in a serial fourfold dilution series, were added in two wells of a flat bottom tissue culture microtiter plate (COSTAR, Corning Incorporated, NY 14831, USA), mixed with an equal volume of 50 $TCID_{50}$ of a SARS-CoV2 strain isolated from a symptomatic patient, previously titrated and incubated at 33°C in 5% CO_2. All dilutions were made in EMEM with addition of 1% penicillin, streptomycin and glutammin and 5 γ/mL of trypsin. After 1 h incubation at 33°C 5%CO_2, VERO E6 cells [VERO C1008 (Vero 76, clone E6, Vero E6); ATCC® CRL-1586™] were added to each well. At 48 h of incubation at 33°C 5%CO_2wells were stained with Gram's crystal violet solution (Merck KGaA, 64271 Damstadt, Germany) plus 5% formaldehyde 40% m/v (Carlo ErbaSpA, Arese (MI), Italy) for 30 min. Microtiter plateswere then washed in running water. Wells were scored to evaluate the degree of cytopatic effect (CPE) compared to the virus control. Blue staining of wells indicated the presence of neutralizing antibodies. Neutralizing titre was the maximum dilution with the reduction of 90% of CPE. A positive titre was equal or greater than 1:10. Positive and negative controls were included in all test run **(Figure S1)**.

In order to define sensitivity and specificity of the novel microneutralization assay, 30 stored sera collected in the period 2011-2013 (negative controls) as well as 40 sera from convalescent patients with real-time RT PCR proven COVID-19 disease were tested.

SARS-CoV-2 diagnosis from nasal swabs

Respiratory samples from upper (FLOQSwabs™, Copan Italia, Brescia, Italy) respiratory tract (bronchialveolar lavage and broncho aspirates) were collected in blood donors. Total nucleic acids (DNA/RNA) were extracted from 200 µl of UTM™ using the QIAsymphony® instrument with QIAsymphony® DSP Virus/Pathogen Midi Kit (Complex 400 protocol) according to the manufacturer's instructions (QIAGEN, Qiagen, Hilden, Germany). Specific real-time RT-PCRs targeting RNA-dependent RNA polymerase and E genes were used to detect the presence of SARS-CoV2 according to the WHO guidelines [2] and Corman and collegues protocols [3].

Data analysis

Descriptive data were reported or considered as absolute and relative frequencies, median and range or interquartil range (IQR) based on the type of the variable distribution.

Supplementary figure

Figure S1

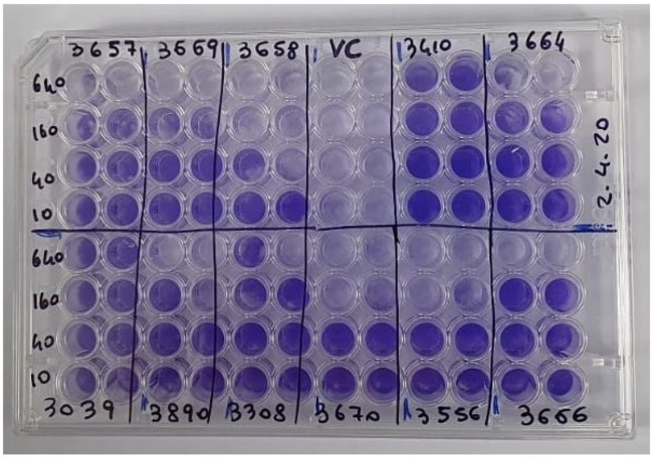

Figure 1S. A picture of exemplificative microneutralization plate is shown. All the samples are tested in duplicate in four-fold scalar dilution (numbers 10, 40, 160 and 640 on the left). Blue staining of wells indicated the presence of neutralizing antibodies; VC, virus control; 4-digit numbers at the top and bottom of the plate are pts identification numbers; 2.4.20 on the right of the plate indicate the reading date (April 2, 2020).

References

1. Percivalle E, Cassaniti I, Sarasini A, Rovida F, Adzasehoun KMG, Colombini I, Isernia P, Cuppari I, Baldanti F. West Nile or Usutu Virus? A Three-Year Follow-Up of Humoral and Cellular Response in a Group of Asymptomatic Blood Donors. Viruses. 2020 Jan 29;12(2):157. doi: 10.3390/v12020157.
2. https://www.who.int/docs/default-source/coronaviruse/protocol-v2-1.pdf
3. Corman VM, Landt O, Kaiser M, Molenkamp R, Meijer A, Chu DK, et al. Detection of 2019 novel coronavirus (2019-nCoV) by real-time RT-PCR. Euro Surveill. 2020;25(3):pii=2000045. https://doi.org/10.2807/1560-7917.ES.2020.25.3.2000045.
